# Survival and prognostic factors for primary lung extranodal NK/T-cell lymphoma: a retrospective study of data from China and the SEER database

**DOI:** 10.3389/fonc.2025.1496735

**Published:** 2025-01-24

**Authors:** Qiuyu Li, Haoyu Zuo, Chengyang Liu, Jing Yang, Nini Dai

**Affiliations:** ^1^ Department of Respiratory and Critical Care Medicine, Peking University Third Hospital, Beijing, China; ^2^ Department of Pathology, Institute of Systems Biomedicine, School of Basic Medical Sciences, Peking-Tsinghua Center for Life Sciences, Beijing Key Laboratory of Tumor Systems Biology, Peking University Health Science Center, Beijing, China; ^3^ Department of Pathology, Peking University Third Hospital, Beijing, China

**Keywords:** Epstein–Barr virus, extranodal NK/T-cell lymphoma, prognostic factors, SEER database, sex

## Abstract

**Background and aim:**

Extranodal NK/T-cell lymphoma (ENKTL) is a rare and aggressive subtype of non-Hodgkin’s lymphoma that most commonly affects the nasal cavity and nasopharynx. The lung is a rare site for ENKTL involvement, and its clinical behavior and prognostic factors are not well understood. This study aimed to analyze survival outcomes and identify prognostic factors in patients with primary lung ENKTL.

**Methods:**

A retrospective analysis was conducted using data from 20 cases of primary lung ENKTL, including four patients who were treated at Peking University Third Hospital in Beijing and 16 patients were extracted from the Surveillance, Epidemiology, and End Results Program database. Clinical characteristics, treatment modalities, and survival data were collected and analyzed using Kaplan–Meier and Cox regression models to identify potential prognostic factors.

**Results:**

The study cohort included 13 male (65%) and 7 female (35%) patients with a median age of 57 years. Sex was a significant predictor of survival (*P* = 0.030), with female patients having lower survival rates. Other factors, including age, race, and disease stage, were not significantly associated with survival. Most patients received chemotherapy (45%) or a combination of chemotherapy and radiotherapy (5%), but treatment data were incomplete for 40% of the cohort. The median overall survival was poor, reflecting the aggressive nature of primary lung ENKTL.

**Conclusions:**

Primary lung ENKTL is a rare, aggressive malignancy with limited available data. In this cohort, sex was a significant prognostic factor, while other demographic and clinical variables did not show significant associations with survival. Future research should focus on understanding the molecular and immunological drivers of this disease, with an emphasis on discovering novel therapeutic approaches. Large-scale multicenter studies are needed to improve diagnostic and treatment strategies for primary lung ENKTL.

## Introduction

Extranodal NK/T-cell lymphoma (ENKTL) is a distinct, relatively rare subtype of T-cell lineage lymphomas that is classified under non-Hodgkin’s lymphoma (NHL). ENKTL accounts for 5% to 10% of all lymphoma cases and approximately 15% of peripheral T-cell lymphomas (1, 2), marking its importance in hematologic oncology.

ENKTL is an aggressive, often fatal disease, with a rapid progression and challenging 5-year overall survival (OS) rate of ~49.5% (3), underscoring the urgent need for effective therapeutic strategies. In more than 80% of patients, ENKTL arises in the nasal cavity and nasopharynx. Although less common, it can also involve extranasal sites such as the skin, soft tissues, testes, and lungs. Primary lung ENKTL is exceptionally rare and not fully understood, as it often presents with non-specific respiratory symptoms that complicate diagnosis (4, 5).

The PD-1/PD-L1 axis is a significant molecular pathway implicated in ENKTL, including lung cases, with PD-L1 overexpression frequently seen, especially in Epstein–Barr virus (EBV)-associated cases. In primary lung ENKTL, PD-L1 overexpression aids tumor cells in evading immune detection, which accelerates disease progression and worsens outcomes (6). EBV-driven LMP1 activates the NF-κB pathway, amplifying PD-L1 expression and facilitating immune escape, particularly in the immune-tolerant lung environment due to ongoing exposure to environmental antigens (7).

Genetic alterations, such as TP53 mutations identified in ENKTL, have been linked to more aggressive disease phenotypes. In primary lung ENKTL, TP53 mutations exacerbate disease progression by impairing apoptosis, leading to uncontrolled cellular proliferation and resistance to conventional therapies (4). These molecular abnormalities are further influenced by the lung’s unique environment, which is characterized by constant exposure to external pathogens, creating a dynamic microbiome marked by specialized immune responses. This distinct pulmonary microenvironment may play a critical role in shaping the molecular and cellular behavior of ENKTL, emphasizing the need for tailored molecular therapies to address these challenges. Given the aggressive and rare nature of primary lung ENKTL, studying its molecular mechanisms within the context of the lung’s specialized environment is vital for advancing diagnostic and therapeutic approaches.

## Methods

### Study population and design

This retrospective, longitudinal study drew on data from two sources, including four patient cases from the Department of Respiratory and Critical Care Medicine at Peking University Third Hospital and 16 patient cases from the Surveillance, Epidemiology, and End Results (SEER) database, a publicly accessible database comprising patient records from 18 state and municipal registries covering approximately 34.6% of the U.S. population.

The inclusion criteria were as follows:

- Histopathological diagnosis consistent with ENKTL based on World Health Organization criteria
- No prior history of lymphoma at other anatomical sites
- Chest imaging exhibiting lung and/or bronchial involvement without mediastinal lymph node enlargement and no other lymphoma or lymphopoietic system tumor at other sites
- Absence of extrapulmonary invasion for at least 3 months post-diagnosis

The study protocol was approved by the Ethics Committee of Peking University Third Hospital (Approval No. IRB00006761-M2022102 2022-P2-137-01 2020KT103). If a patient was admitted to the Respiratory and Critical Care Medicine Unit multiple times, only data from the initial admission were incorporated into the analysis.

### Data collection

The patient data, which was collected from medical records, included demographic details such as age, sex, clinical symptoms, allergies, family history, primary tumor characteristics, tumor location, pathologic stage, and survival. The associations between sex, age, race, pathologic stage, and patient survival were analyzed. Follow-up data for patients from our institutional cohort (Peking University Third Hospital) were collected via telephone interviews. Associations between sex, age, smoking status, tumor differentiation, tumor size, lymph node metastasis, pathologic stage, as well as patient survival were analyzed. Patients were followed up at 3-month intervals following discharge from the hospital to monitor survival, recurrence, treatment changes, and treatment-related complications. Follow-up data for patients identified from the SEER database were extracted from the database (8).

### Survival analysis

This research primarily focused on cancer-specific survival (CSS), which is a net survival in the absence of other causes of death and refers to its categorization of mortality cause, as documented in the patients’ medical records or data extracted from the SEER database. The survival time was calculated from the date of initial diagnosis to the date of death, or until the date of censoring for patients who were still alive at the last follow-up. CSS is an important metric that reflects the likelihood of surviving a specific cause of death while excluding the influence of other causes of mortality.

### Statistical analysis

The data were analyzed using SPSS 27.0 software (IBM, New York, USA). Continuous variables were expressed as the means ± standard deviations, whereas categorical variables were presented as frequencies and proportions. One-way analysis of variance was used to assess the significance of the mean differences across categories, while the chi-squared test or Fisher’s exact test was used for categorical variables. All *P*-values were two-sided, and a *P*-value of less than 0.05 was considered to indicate statistical significance.

Kaplan–Meier analysis was performed to analyze the categorical variables, and a univariate Cox regression model was employed for continuous variables. This comprehensive statistical examination of the variables highlighted those that exhibited the most robust associations and predictive capabilities.

## Results

### Demographic and clinical characteristics of the participants

A total of 20 patients with primary lung ENKTL met the criteria for inclusion in this study. The patient cohort comprised four individuals treated at the Department of Respiratory and Critical Care Medicine at Peking University Third Hospital and 16 patients whose records were obtained from the SEER database. Of the 20 patients, 13 were male (65%) and seven were female (35%), with a median age of 57 years (25 to 88 years). Of the cohort, eight patients (40%) were older than 60 years, while 12 patients (60%) were 60 years of age or younger at the time of diagnosis. Nine patients (45%) identified as White, one patient (5%) identified as Black, and the remaining 10 patients (50%) were categorized under other racial groups.

The Ann Arbor-Cotswold staging system was used to classify the extent of the disease, with three patients (15%) assigned to stages I–II and five patients (25%) assigned to stages III–IV, while the remaining 12 patients (60%) could not be staged due to incomplete data collection. All 20 patients had ENKTL involving the lungs; primarily originating in the lungs, whereas in two patients (10%), the lymphoma originated elsewhere and subsequently metastasized to the lungs. These two patients were included based on detailed pathological, imaging, and clinical assessments. Although their lymphoma originated outside the lungs, the primary site of disease progression and most significant clinical involvement were centered in the lungs. The lack of substantial involvement in the originally affected site upon diagnosis and during follow-up supports their classification as having predominant lung involvement. As such, we considered their clinical trajectory as highly relevant to understanding the prognosis and treatment outcomes of primary lung ENKTL. To ensure the robustness of our findings, we conducted sensitivity analyses excluding these two cases. The key results, including the identification of prognostic factors, remained consistent, suggesting that their inclusion did not introduce significant bias or alter the main conclusions of the study.

B symptoms, including systemic manifestations such as fever, unexplained weight loss, and night sweats, are often associated with a worse prognosis in lymphoma cases. In this cohort, nine patients (45%) had B symptoms, one patient (5%) did not exhibit B symptoms, and 10 patients (50%) had inconclusive data regarding the presence of B symptoms (50%).

Regarding the treatment they received, two patients (10%) received radiation therapy alone, nine patients (45%) underwent chemotherapy, and one patient (5%) received a combination of both radiotherapy and chemotherapy. The treatment modalities for the remaining eight patients (40%) were not documented, which reflects the inherent limitations of retrospective studies, where complete treatment records may not always be available.

A detailed summary of the clinical data for all patients can be found in **Table 1**.

**Table 1:** Basic information about NK/T lymphoma patients.

Variables	Total (n=20)
Age, n (%)	
>60	8 (40%)
≤60	12 (60%)
Race	
White	9 (45%)
Black	1 (5%)
Others	10 (50%)
Sex	
Male	13 (65%)
Female	7 (35%)
Primary Site	
Lower lobe	2 (10%)
Middle lobe	1 (5%)
Upper lobe	4 (20%)
Overlapping lesion of lung	3 (15%)
Lung, NOS	10 (50%)
B symptoms	
Yes	9 (45%)
No	1 (5%)
Unknown	10 (50%)
Ann Abor stage	
Stage I–II	3 (15%)
Stage III–IV	5 (25%)
Unknown	12 (60%)
Therapy	
Radiation	2 (10%)
Chemotherapy	9 (45%)
Combined radiation and chemotherapy	1 (5%)
Unknown	8 (40%)

### Survival analysis

Kaplan–Meier survival analyses, in conjunction with Cox regression models, were employed to assess the potential prognostic factors that could influence survival outcomes in patients with primary lung ENKTL. The variables examined included age, sex, race, and Ann Arbor stage classification. The univariate analysis indicated a statistically significant impact of sex on survival (*P* = 0.030), with notable differences between male and female patients. However, no statistically significant associations were found between survival and other variables, such as age, race, or Ann Arbor stage classification. These results suggested that, in our cohort, sex played a prominent role in determining survival outcomes compared to other commonly considered factors.

**Figure 1:**
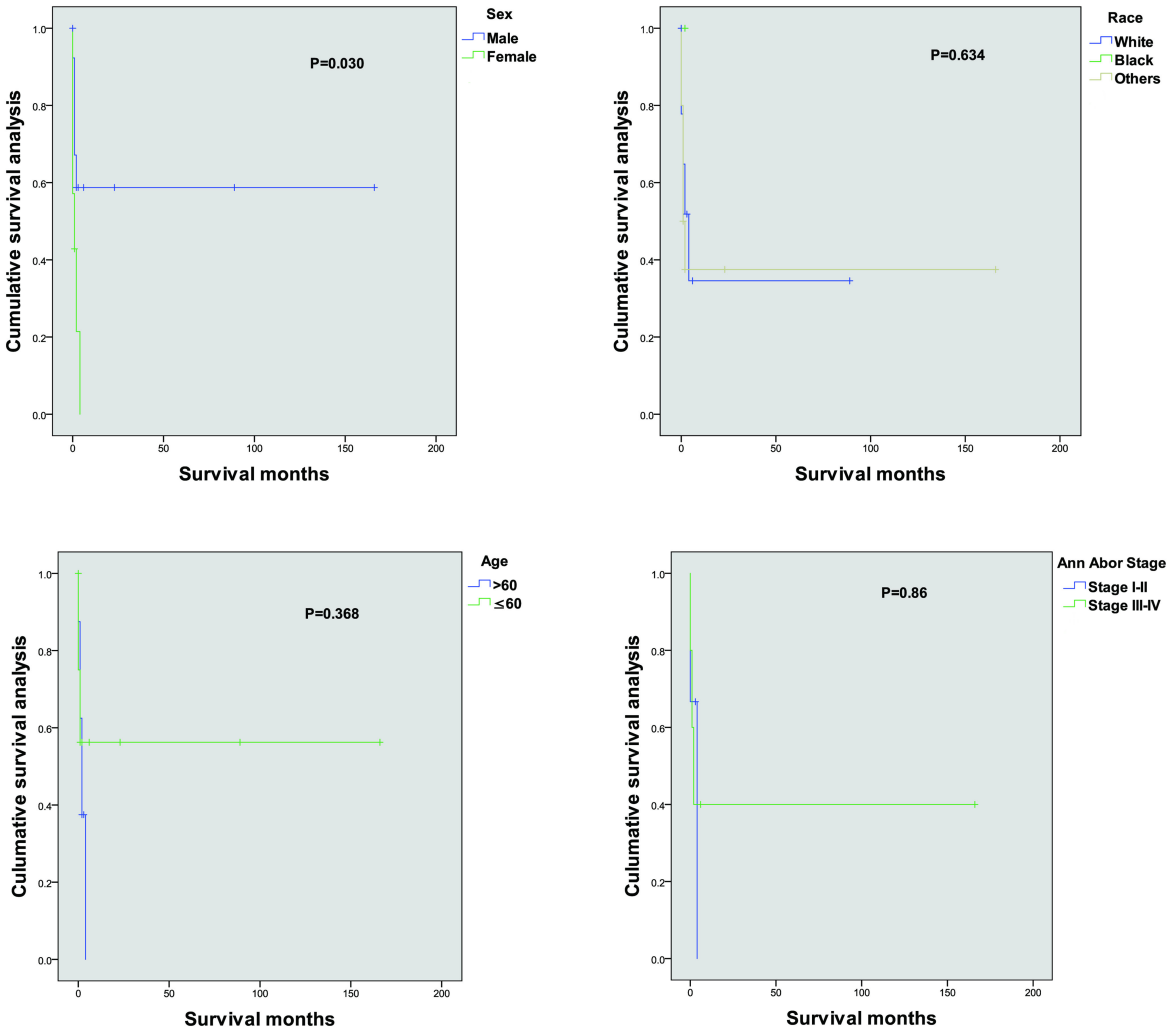
Kaplan–Meier cancer-specific survival curves for patients according to different independent prognostic factors.

The Kaplan–Meier survival curves corresponding to each independent prognostic factor are presented in **Figure 1**. The presented curves illustrate the survival trends across distinct patient subgroups, thereby highlighting the impact of sex on OS in this specific cohort of primary lung ENKTL patients.

### Case report: a rare case of primary pulmonary EKNTL

A representative case of primary ENKTL demonstrates the techniques used for evaluation of the patient and the relevant findings implicated in specific treatment responses. The selected case is illustrative of the principal pathological features and imaging patterns observed in our patient cohort and aligns with those reported in the existing literature on primary lung ENKTL, thus providing a reference point for the unique aspects of this disease subtype.

#### Patient background

A 67-year-old male presented with a chief complaint of cough and shortness of breath for one month. The symptoms were intermittent and included occasional yellow sputum production, chest discomfort in the right anterior rib region, and night sweats. There was no fever, hemoptysis, back pain, or other systemic symptoms. The patient reported a smoking history of 30 years and hypertension managed with valsartan 80 mg daily.

### Pathological examination

**Figure 2:**
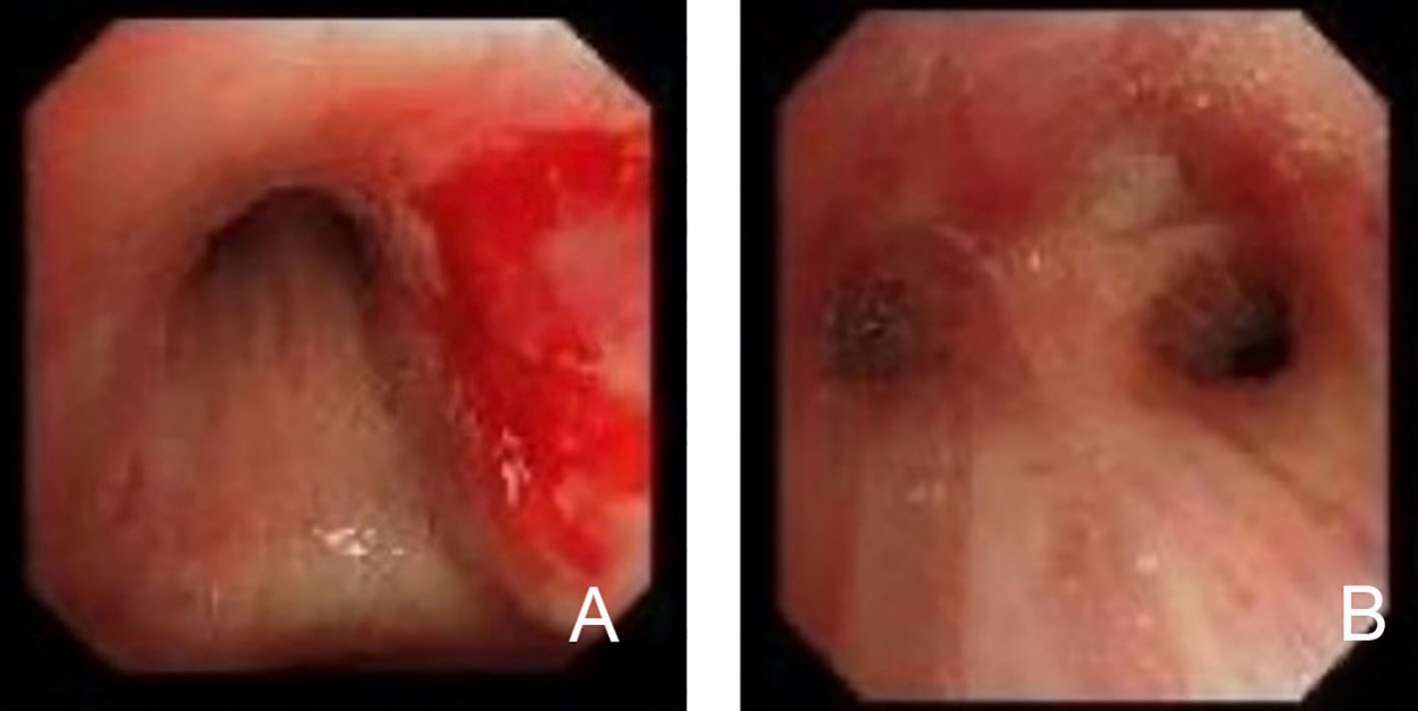
Bronchoscopic findings in primary lung extranodal NK/T-cell lymphoma (ENKTL). **(A)** Bronchoscopic view of the right bronchus, showing diffuse mucosal erythema, swelling, and hemorrhagic lesions. The mucosa appears significantly thickened and inflamed, contributing to the narrowing of the bronchial lumen. **(B)** Bronchoscopic view demonstrating focal areas of necrosis and ulceration in the bronchial mucosa, typical of NK/T-cell lymphoma involvement. The bronchial lumen is further constricted due to the infiltration of malignant cells and associated tissue damage. These bronchoscopic findings highlight the characteristic mucosal involvement in primary lung ENKTL, including mucosal thickening, hemorrhage, and necrosis, contributing to airway obstruction and respiratory symptoms.

Bronchoscopy under local anesthesia revealed notable pathological findings in the right upper lung bronchus (**Figure 2**). The bronchial mucosa was diffusely congested, edematous, and thickened, with significant narrowing of the lumen, particularly in the anterior segment. A biopsy was performed on the anterior segment of the right upper lobe, and pathological examination showed squamous epithelial metaplasia accompanied by substantial infiltration of lymphocytes and neutrophils. Focal squamous epithelial hyperplasia with areas of multifocal necrosis was also observed.

**Figure 3:**
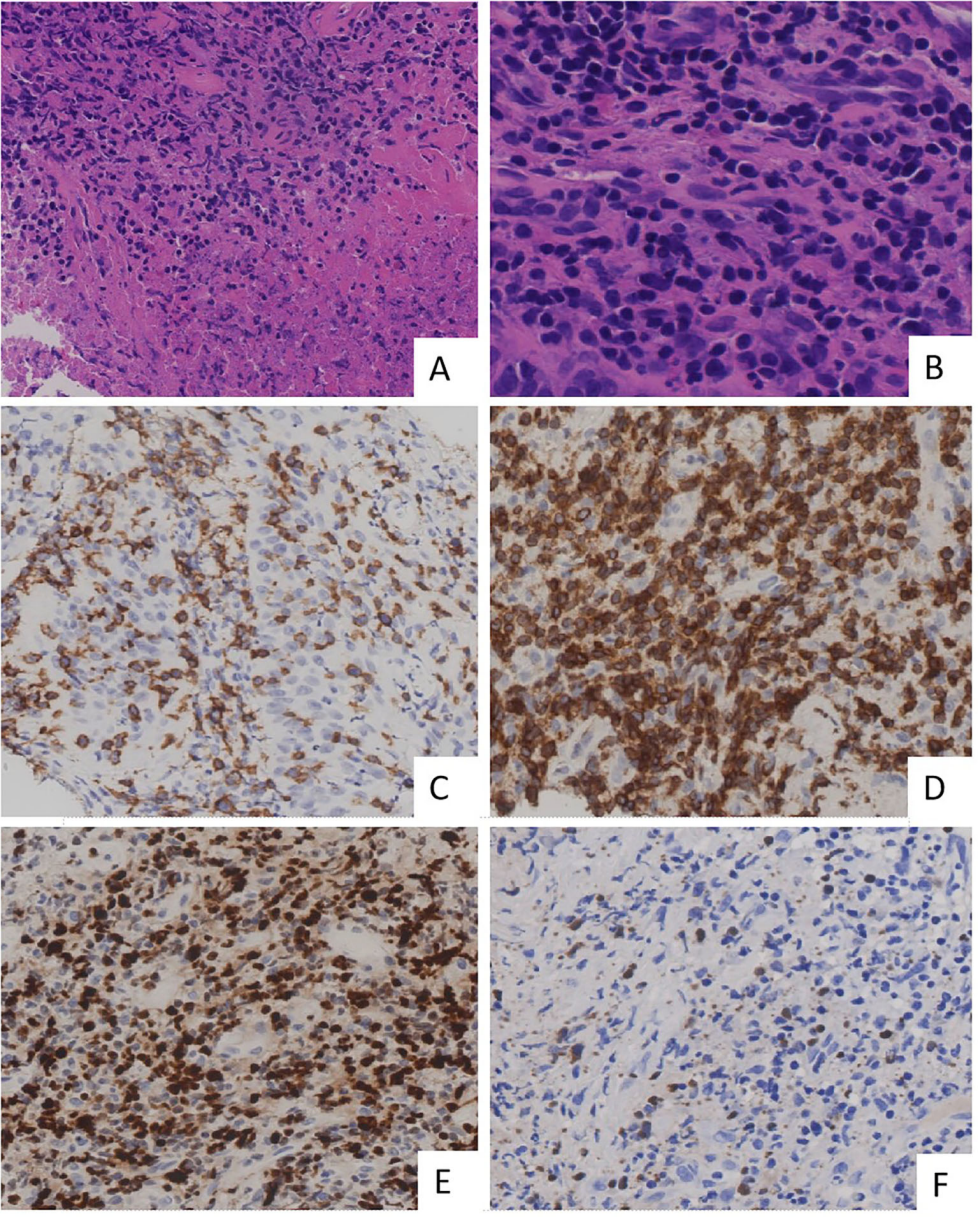
Histopathological and immunohistochemical findings in primary lung extranodal NK/T-cell lymphoma. **(A)** H&E staining demonstrates extensive necrosis with a dense infiltrate of atypical lymphoid cells. **(B)** Height magnification of H&E staining shows the lymphoma is composed of medium-sized cells. These cells exhibit irregularly folded nuclei, and nucleoli are inconspicuous. **(C)** Immunohistochemical staining for CD56 shows strong membrane positivity in neoplastic cells, confirming NK cell lineage. **(D)** Immunostaining for CD3 is positive, consistent with the presence of T cells, further supporting the diagnosis of NK/T-cell lymphoma. **(E)** Ki-67 proliferation index shows a high percentage of positive cells, indicating a highly proliferative tumor with aggressive behavior. **(F)** EBER *in situ* hybridization shows positive cells, confirming the presence of EBV infection within these cells. These findings collectively confirm the diagnosis of primary lung ENKTL, characterized by high-grade lymphoid infiltration and strong expression of NK/T-cell markers. EBER, Epstein–Barr virus-encoded RNA; ENKTL, extranodal NK/T-cell lymphoma; H&E, hematoxylin and eosin; NK, natural killer.

The immunohistochemistry test revealed the following: TTF-1 (mucosal+), Napsin A (-), P40 (basal cells+), CK5/6 (mucosal+), CgA (-), Syn (-), CD56 (+), CD20 (scattered+), CD3 (+), CD21 (-), and Ki-67 (50%+) (**Figure 3**). The special staining test showed PAS (-), PASM (-), and acid-fast staining (-). The molecular pathology test of a fluorescent polymerase chain reaction for the detection of *Mycobacterium tuberculosis* was negative, whereas *in situ* hybridization for EBV-encoded RNA was positive. Considered together, these findings strongly supported the ENKTL lymphoma diagnosis.

### Imaging characteristics

**Figure 4:**
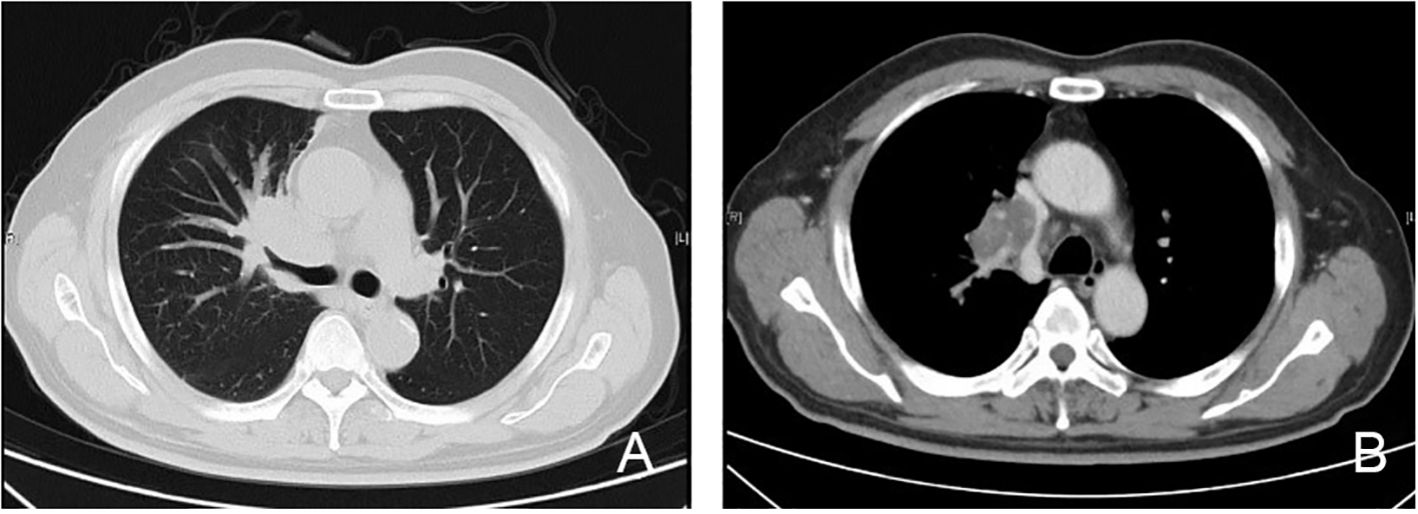
CT imaging of primary lung extranodal NK/T-cell lymphoma (ENKTL). **(A)** Lung window CT scan shows an irregular mass in the right hilum, with evidence of airway compression and partial obstruction of the bronchus. The mass is lobulated with spiculated borders, consistent with an aggressive lesion, and appears to be close in proximity to the pulmonary vasculature. **(B)** Mediastinal window CT scan demonstrates enlargement of several mediastinal lymph nodes, suggesting possible lymphatic involvement. The mass appears to be invading adjacent mediastinal structures, raising concerns about local invasion into the surrounding tissues and vascular compression. CT, computed tomography; ENKTL, extranodal NK/T-cell lymphoma; NK, natural killer.

**Figure 5:**
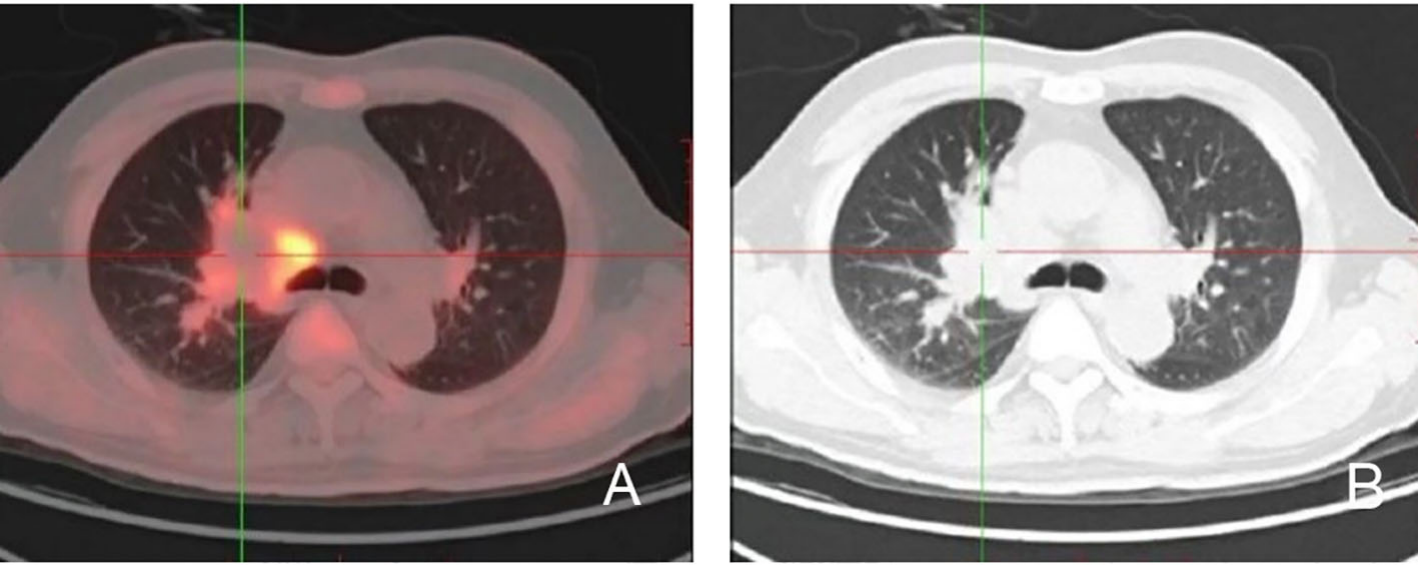
PET-CT imaging of primary lung extranodal NK/T-cell lymphoma. **(A)** The CT component of the PET-CT scan shows an irregular mass in the right hilum, adjacent to the pulmonary vessels, with evidence of local invasion and airway compression. The lesion is characterized by irregular borders and lobulated appearance. **(B)** The fused PET-CT image reveals intense FDG uptake in the same right hilar mass, indicating hypermetabolic activity, consistent with a malignant process. The high SUV suggests aggressive tumor behavior. Increased FDG uptake is also visible in the surrounding tissue, raising concerns for possible lymphatic or adjacent structure involvement. CT, computed tomography; ENKTL, extranodal NK/T-cell lymphoma; FDG, fluorodeoxyglucose; PET-CT, positron emission tomography; NK/T-cell lymphoma; NK, natural killer; SUV, standardized uptake value.

Several imaging modalities were employed to assess the extent of the disease. A computed tomography (CT) scan (**Figure 4**) showed that the mass located in the right hilum caused airway narrowing and distortion of adjacent lung structures. The mass exhibited a lobulated appearance with spiculated borders, further suggesting an aggressive tumor. There was evidence of possible invasion into nearby mediastinal structures, although this required further evaluation to confirm the extent of invasion. A positron emission tomography-CT (PET-CT) scan (**Figure 5**) revealed significant fluorodeoxyglucose (FDG) uptake within the right hilar mass, indicating hypermetabolic activity consistent with malignancy. The high standardized uptake value of the lesion supported the possibility of an active tumor. Involvement of adjacent structures, including potential lymphatic spread to the mediastinum, was evident, as there was increased FDG uptake in the region, suggesting lymphatic involvement.

## Discussion

ENKTL is a rare and aggressive form of NHL that primarily occurs outside the lymph nodes. It is typically characterized by extensive necrosis, vascular destruction, and an association with EBV (9). ENKTL most frequently originates in the nasal cavity, where it is referred to as nasal-type ENKTL, and the occurrence of primary lung ENKTL is exceedingly rare. Due to its rarity, non-specific clinical manifestations, and frequent challenges it poses for obtaining adequate biopsy tissue, primary lung ENKTL diagnoses are often delayed or missed. Additionally, prognostic factors for this specific variant are poorly understood and the subject of ongoing debate.

The age of onset for ENKTL spans a wide range, from as young as 9 years to as old as 89 years, although the disease is predominantly seen in adults, with a median age ranging from 46 to 60 years in most studies. A notable feature of ENKTL is its male predominance, with males representing 55% to 78% of cases (3). Moreover, ENKTL is more frequently diagnosed in East Asian populations, and to a lesser extent in Central and South Americans, suggesting potential geographical and ethnic predispositions. Despite this, the underlying mechanism driving these epidemiological trends remains elusive. However, EBV infection has been a key factor linking ENKTL across different ethnic and geographical groups (4). EBV plays a central role in the pathogenesis of ENKTL, potentially influencing its onset and progression.

In the current study, Kaplan–Meier survival analysis and Cox regression models identified sex as a significant prognostic factor in patients with primary lung ENKTL, with female patients exhibiting lower survival rates than male patients (*P* = 0.030). Although male patients generally have worse outcomes in many types of cancer due to their higher exposure to risk factors like smoking, occupational hazards, and higher overall cancer incidence, this unexpected finding that female patients have lower survival in primary lung ENKTL may be attributed to several biological and clinical factors that are unique to female patients.

First, the poorer prognosis observed in female patients may have been linked to hormonal differences influencing the tumor microenvironment dynamics and immune responses. While estrogen is generally considered immunosuppressive, its effects are context-dependent and vary based on the disease and tumor type. Studies have demonstrated that estrogen modulates the expression of key cytokines, such as interleukin-6 and tumor necrosis factor-alpha, which contribute to tumor progression and immune evasion in some cancers, including lymphomas. Additionally, the interplay between estrogen signaling and EBV activity—which is central to the pathogenesis of ENKTL—may exacerbate disease progression in female patients, as estrogen has been implicated in modulating EBV lytic cycle activation (1). Furthermore, sex-related differences in treatment response may have contributed to the survival disparity. For instance, females may metabolize chemotherapeutic agents differently due to variations in hepatic enzyme activity, thus impacting drug efficacy and toxicity profiles. These pharmacokinetic and pharmacodynamic differences might have partially contributed to the poorer survival outcomes observed in female patients.

Second, hormonal differences may contribute to sex-specific tumor biology in ENKTL. This could create an immunosuppressive environment that facilitates uninhibited tumor growth, particularly in EBV-driven ENKTL, where immune evasion is already a hallmark of disease progression.

Estrogen plays a key role in modulating the immune system, influencing both NK and T-cell functions. Estrogen affects NK cell cytotoxicity, with evidence showing that it can enhance NK cell function by increasing the expression of activating receptors like NKG2D (10, 11). However, the effect of estrogen on NK cells is context-dependent, as estrogen may also impair NK cell-mediated cytotoxicity in certain autoimmune conditions (12). The complex role of estrogen in NK cells may explain some of the observed differences in immune response and tumor progression, particularly in female patients with ENKTL.

Regarding T-cells, estrogen can influence both CD4+ and CD8+ T-cells. Estrogen enhances the differentiation of T helper 2 (Th2) cells, which are involved in promoting antibody production and immune responses to extracellular pathogens (13). However, estrogen may also inhibit CD8+ cytotoxic T-cells, which are crucial for eliminating tumor cells. Estrogen suppresses CD8+ T-cell activity by reducing the production of interferon-gamma, thereby dampening their anti-tumor functions. Additionally, estrogen promotes the expansion of regulatory T-cells (Tregs), which suppress immune responses and may facilitate immune evasion by the tumor (14, 15).

Furthermore, estrogen’s influence on the tumor microenvironment in female patients could have contributed to the observed differences in prognosis (16). Estrogen can promote angiogenesis, which facilitates tumor growth and metastasis. Additionally, estrogen affects cellular interactions within the tumor stroma, which can alter the tumor’s immunological environment, thus supporting tumor survival and growth (17). These factors may collectively explain the poorer outcomes observed in female patients with primary lung ENKTL.

Finally, it is possible that treatment-related factors play a role in the observed survival differences between sexes. Previous research has demonstrated that females often experience different pharmacokinetics and pharmacodynamics compared to males, which can affect drug metabolism and response to chemotherapy (5). This could result in less effective treatment outcomes or increased toxicity, potentially contributing to the lower survival rates seen in female patients. Additionally, differences in treatment adherence, healthcare access, and social support systems may also disproportionately affect female patients, further influencing their prognosis (18).

The lower survival rates observed in female patients with primary lung ENKTL may be the result of complex interactions between immune system differences, hormonal influences, and treatment responses. Further studies are needed to explore these mechanisms in greater depth, with an emphasis on understanding how these factors can be leveraged to improve outcomes for female patients.

Additionally, there is an urgent need for future experimental models and molecular-level studies to verify the biological mechanisms attributing to gender differences in immune function. Specifically, further research is needed to elucidate how estrogen modulates the immune system at the molecular level, particularly in its impact on NK and T-cell functionality in both the tumor microenvironment and systemic immunity. These studies will be crucial for developing targeted therapeutic strategies that can account for sex-based differences in immune response and improve treatment outcomes for patients with ENKTL and other malignancies.

An additional finding of our study is the lack of significant impact of disease stage on patient survival, which may be attributed to the intrinsic biological characteristics of NK/T-cell lymphoma. Unlike many other cancers in which the anatomical stage at diagnosis is a critical determinant of prognosis, the survival outcomes in NK/T-cell lymphoma are more closely tied to molecular and genetic factors. This includes EBV infection and mutations in genes such as TP53 and PD-L1, which not only drive aggressive disease progression but also contribute to immune system evasion (6, 7).

Interestingly, even patients diagnosed at an early stage can have poor outcomes if their tumors exhibit aggressive molecular features. This observation highlights the limited prognostic utility of conventional staging systems in NK/T-cell lymphoma, as tumor biology and treatment response are likely to be more important determinants of survival (8). Studies have consistently shown that response to therapy, particularly chemoradiotherapy, is a stronger predictor of survival than the extent of disease at diagnosis. This underscores the critical need for integrating molecular and immunological markers into prognostic models to better guide treatment decisions and improve patient outcomes (19).

Despite these insights, our study is not without limitations. First, the relatively small sample size of 20 patients inevitably limits the statistical power of our findings. Although this is a consequence of the rarity of primary lung ENKTL, it nonetheless reduces the generalizability of our conclusions. Larger, multicenter studies are necessary to confirm our observations and provide a more comprehensive understanding of the disease. Second, the retrospective nature of this study introduces potential biases, such as incomplete or missing data, which could have influenced our analyses.

Specifically, the lack of detailed treatment records for a significant proportion of patients may have impacted the interpretation of treatment efficacy and survival outcomes. Patients for whom treatment data was not recorded could have received different therapeutic interventions, potentially skewing the survival data. Without a full understanding of the treatments administered, it is difficult to draw accurate conclusions regarding the efficacy of specific therapeutic approaches. Additionally, missing data on treatment modalities could have introduced confounding variables that we were unable to account for in our analyses, thus further limiting the robustness of our results. Future studies should aim to collect more comprehensive treatment data to ensure a clearer understanding of the relationship between therapeutic interventions and patient outcomes.

Our study contributes to the growing body of literature on primary lung ENKTL, shedding light on some of the key clinical and demographic characteristics of this rare and aggressive lymphoma. The findings highlight the importance of molecular and immunological factors in shaping prognosis, underscoring the need for novel therapeutic approaches that target these underlying mechanisms.

Future research should place a greater emphasis on investigating molecular targets, particularly those involved in EBV-driven pathways such as LMP1 and the PD-L1 axis. These pathways are critical in the pathogenesis and immune evasion mechanisms of ENKTL, and targeting them may hold the key to more effective treatments. Larger, multicenter studies are needed to validate these findings and assess the therapeutic potential of inhibiting these molecular pathways in a broader patient population.

Additionally, there is a growing need to develop personalized treatment strategies, especially those that consider sex-based differences in immune response and tumor biology. Immunomodulatory therapies tailored to the specific biological profiles of male and female patients may help improve survival rates and outcomes across different patient subgroups. By integrating insights from molecular research with personalized approaches, future therapeutic strategies could more precisely address the complexities of primary lung ENKTL, ultimately leading to improved patient care and prognosis.

## Conclusion

In conclusion, primary lung ENKTL is a rare and aggressive form of NHL, with distinct clinical and pathological features. Our retrospective analysis, based on data from Peking University Third Hospital and the SEER database, highlights the diagnostic and therapeutic challenges posed by its rarity and non-specific presentation. Despite the use of chemotherapy and radiotherapy, the overall prognosis remains poor, particularly with sex identified as a significant predictor of survival, while age, race, and Ann Arbor staging were not. The limited sample size precludes the formulation of broader conclusions, reflecting the inherent difficulty of studying a disease with such a low prevalence.

Further research should focus on understanding the molecular and immunological mechanisms underlying primary lung ENKTL, particularly its association with EBV. Targeted therapies and immunomodulatory approaches show promise, and multicenter collaborations are essential for advancing the diagnosis, treatment, and outcomes of this rare malignancy.

## Data Availability

The datasets presented in this study can be found in online repositories. The names of the repository/repositories and accession number(s) can be found in the article/**Supplementary Material**.
